# Tribology of MXene Materials: Advances, Challenges, and Future Directions

**DOI:** 10.3390/ma18204767

**Published:** 2025-10-17

**Authors:** Jonathan Luke Stoll, Mason Paul, Lucas Pritchett, Ashleigh Snover, Levi Woods, Subin Antony Jose, Pradeep L. Menezes

**Affiliations:** Department of Mechanical Engineering, University of Nevada-Reno, Reno, NV 89557, USA; jlstoll117@gmail.com (J.L.S.); masonpaul@unr.edu (M.P.); lpritchett@unr.edu (L.P.); leviwoods@unr.edu (L.W.); subinj@unr.edu (S.A.J.)

**Keywords:** MXene synthesis, MXene terminations, tribology, wear, composites

## Abstract

MXenes, an emerging class of two-dimensional (2D) transition metal carbides, nitrides, and carbonitrides, have demonstrated exceptional potential in tribology: the study of friction, wear, and lubrication. Their remarkable mechanical strength, thermal stability, and tunable surface chemistry make them ideal candidates for solid lubricants, lubricant additives, and protective coatings in mechanical systems. This review comprehensively examines the tribological performance of MXenes under diverse environmental conditions, including high temperatures, vacuum, humid atmospheres, and liquid lubricants. A particular emphasis is placed on the influence of surface terminations (-OH, -O, -F) on friction reduction and wear resistance. Additionally, we discuss strategies for enhancing MXene performance through hybridization with polymers, nanoparticles, and ionic liquids, enabling superior durability in applications ranging from micro/nano-electromechanical systems (MEMS/NEMS) to aerospace and biomedical devices. We also highlight recent advances in experimental characterization techniques and computational modeling, which provide deeper insights into MXene tribomechanics. Despite their promise, key challenges such as oxidation susceptibility, high synthesis costs, and performance variability hinder large-scale commercialization. Emerging solutions, including eco-friendly synthesis methods and optimized composite designs, are explored as pathways to overcome these limitations. Overall, MXenes represent a transformative avenue for developing next-generation tribological materials that combine high efficiency, sustainability, and multifunctionality. Continued research and innovation in this field could unlock groundbreaking advancements across industrial and engineering applications.

## 1. Introduction

### 1.1. Overview of MXenes

MXenes are an emerging family of two-dimensional (2D) materials that have garnered significant attention in engineering due to their exceptional mechanical properties, including high strength, ductility, and durability. First discovered in 2011 by researchers at Drexel University [1], MXenes are derived from MAX phases: layered ternary compounds with the general formula M_n_₊_1_AX_n_, where M is a transition metal (e.g., Ti, V, Nb), A is a group 13 or 14 element (e.g., Al, Si), and X is carbon or nitrogen [1,2]. The synthesis of MXenes involves selectively etching the “A” layer from the MAX phase, typically using hydrofluoric acid (HF), which exposes stacked transition metal carbide or nitride layers with surface terminations such as -OH, -O, or -F [3]. “MXene” reflects their structural resemblance to graphene while acknowledging their MAX phase origin. To improve safety and scalability, alternative etching methods, such as the use of lithium fluoride (LiF) and hydrochloric acid (HCl), have been developed to generate in situ HF, facilitating controlled delamination [1]. Additionally, bottom-up approaches like chemical vapor deposition (CVD) are being explored to produce high-quality, large-area MXene films. Since their discovery in 2011, more than 40 distinct MXene compositions have been synthesized, expanding their potential applications across material science and engineering [3].

MXenes exhibit a unique combination of metallic conductivity (up to ~34,500 S/cm) [4] and mechanical robustness (e.g., Young’s modulus of ~484 GPa for Ti_3_C_2_T_x_), making them suitable for energy storage, electromagnetic shielding, and water purification [5]. Recently, their potential in tribology, the study of friction, wear, and lubrication, has gained traction. Like conventional solid lubricants (e.g., MoS_2_, graphite), MXenes exhibit low interlayer shear strength due to weak van der Waals interactions between their functionalized layers, enabling low friction between layers [6]. Beyond lubrication, MXenes offer superior wear resistance, low thermal conductivity [7], and tunable surface chemistry [6], which can be tailored to enhance properties such as corrosion resistance, biocompatibility, and interfacial adhesion. These properties make MXenes promising for advanced tribological applications, including protective coatings, lubricant additives, and components in microelectromechanical systems (MEMS), cutting tools, biomedical implants, and aerospace mechanisms.

The global energy cost of friction underscores the urgent need for advanced lubricants and surface materials. According to Holmberg and Erdemir [8], approximately 20% of the world’s total primary energy consumption is expended in overcoming friction, with an additional 3% lost to wear-related replacement and repair processes. These losses correspond to nearly one-fifth of global energy use, equivalent to hundreds of exajoules annually, highlighting the immense potential impact of efficient lubrication strategies. Developing novel, high-performance lubricants that can mitigate frictional energy losses is therefore a critical objective in materials engineering. MXenes, with their tunable surface chemistry and low shear resistance, represent a promising pathway toward achieving these energy efficiency gains.

MXenes represent a versatile and rapidly evolving class of 2D materials with exceptional electrical, mechanical, and surface properties. Their ease of synthesis, compositional diversity, and scalability position them as next-generation solutions for tribological challenges. As research progresses, precise control over MXene structures and hybrid designs is expected to unlock further breakthroughs in high-performance, energy-efficient, and environmentally sustainable materials for demanding mechanical systems.

### 1.2. Scope and Objectives of the Review

Tribology plays a fundamental role in enhancing the performance, durability, and energy efficiency of engineering systems. By studying surface interactions in relative motion, tribologists seek to minimize energy losses and material degradation caused by friction and wear. Friction arises from the resistance between sliding surfaces, leading to inefficiencies and mechanical failure if unmanaged [9]. Wear, the progressive loss of material due to mechanical contact, further compromises component lifespan. To combat these challenges, researchers employ advanced lubrication strategies, high-performance coatings, and novel material designs. Recent advancements, including machine learning and computational modeling, have further refined the understanding of tribological phenomena such as fretting wear and frictional mechanisms, accelerating innovation in the field [10].

Material selection is critical in tribology, as friction and wear directly impact safety, efficiency, and operational costs across industries such as transportation, manufacturing, and energy. Engineers leverage tribological principles to design optimized surfaces, lubricants, and composites that reduce energy consumption and extend service life [11]. Emerging technologies like additive manufacturing (AM) highlight the growing importance of tribology, as surface roughness and post-processing remain key challenges in processes like laser powder bed fusion (LPBF). Tribological research is addressing these limitations through advanced coatings, nanomaterial-enhanced lubricants, and surface engineering techniques, enabling broader industrial adoption of AM and other cutting-edge technologies [12].

MXenes have emerged as a transformative class of materials in tribology due to their unique 2D structure, exceptional mechanical strength, and tunable surface chemistry. However, despite their promise as solid lubricants and lubricant additives, their tribological behavior requires further exploration [13]. This review provides a comprehensive analysis of MXenes’ role in friction and wear reduction, addressing their advantages, limitations, and prospects. A key focus is the influence of surface terminations (-OH, -O, -F) on MXenes’ tribological performance. These functional groups help dictate crucial material properties like adhesion, shear strength, and environmental adaptability [14]. Such tunability allows MXenes to outperform conventional solid lubricants like MoS_2_ and graphite, remaining applicable under various operating conditions.

This review also evaluates MXenes’ viability as lubricant additives. Experimental studies demonstrate that MXene nanosheets dispersed in oils can reduce friction coefficients by up to 31% when using a 5 wt.% concentration [15]. Their layered structure facilitates low interlayer shear strength, akin to traditional solid lubricants, but with superior thermal and chemical stability. Computational studies further reveal that weak van der Waals forces between MXene layers enable efficient lubrication even under extreme pressures and temperatures [16]. Challenges such as oxidation susceptibility and scalability can hinder adoption under some circumstances. While MXenes can undergo hydrolysis in aqueous environments or tribo-induced oxidation at elevated local temperatures, their ambient air stability has been shown to be excellent in modern high-quality thin films [17]. Potential solutions, including protective coatings, hybrid composites, and green synthesis routes, are critically examined to bridge the gap between laboratory research and commercial applications. By compiling recent advancements in MXene research, this review aims to guide future research toward optimizing MXenes for real-world tribological applications. Their potential spans aerospace, biomedical devices, and energy systems, where their combination of mechanical robustness, thermal stability, and adaptive lubrication could revolutionize material design. Addressing current limitations in stability and manufacturability will be pivotal in unlocking MXenes’ full potential, paving the way for next-generation, sustainable tribological solutions.

### 1.3. Tunability of MXenes for Lubricant Additive Applications

The layered structure and rich surface chemistry of MXenes make them particularly suited for use as lubricant additives in greases and oils. Their surface terminations (such as -O, -OH, -F) and the ability to graft functional molecules allow fine-tuning of both their dispersion behavior and their interfacial interactions with metal surfaces. For instance, DDP-functionalized Ti_3_C_2_T_x_ nanosheets in a 500SN base oil exhibited significantly improved dispersibility and achieved marked reductions in friction and wear compared to unmodified MXene additives [18]. Similarly, surface-functionalized Ti_3_C_2_T_x_ MXene added to base oil delivered friction coefficient reductions of up to ~46% and wear volume reductions exceeding 80% [19].

Beyond friction and wear reduction, MXene additives also enable enhancements in lubricant thermal conductivity, viscosity stability, and load-carrying capacity when properly tuned. A study of Ti_3_C_2_T_x_ as an oil additive showed ~30% friction reduction under high loads and elevated temperatures. A comprehensive review highlights that effective tunability of MXenes (via surface functionalization, flake size control, concentration, and hybridization) is key to their performance as friction modifiers and anti-wear agents in lubricants across different formulations. This tunability offers a clear advantage over conventional solid additives (e.g., MoS_2_, graphite) and opens pathways for designing next-generation additive systems optimized for specific base oils, greases, and operating conditions. Hence, when discussing MXenes in tribological applications, attention to their surface chemistry and additive design is critical for achieving industrially relevant performance.

## 2. Structure and Properties of MXenes

### 2.1. Synthesis and Chemical Composition

MXenes represent a class of two-dimensional transition metal carbides and nitrides with the general formula M_n_₊_1_X_n_T_x_, where M denotes an early transition metal (e.g., Ti, Nb, V, Ta, Mo, or Zr), X is carbon or nitrogen, and T_x_ represents surface functional groups (e.g., -OH, -F, -O) that form during synthesis [20]. These materials are derived from MAX phase precursors (M_n_₊_1_AX_n_) through selective etching of the A layer (usually aluminum or silicon), which creates stacked 2D sheets with functionalized surfaces [21]. The etching process generates charged surfaces that facilitate strong in-plane bonding through van der Waals interactions while maintaining weak interlayer shear strength, a combination that enables excellent lubricating properties through controlled interlayer sliding.

**Figure 1:**
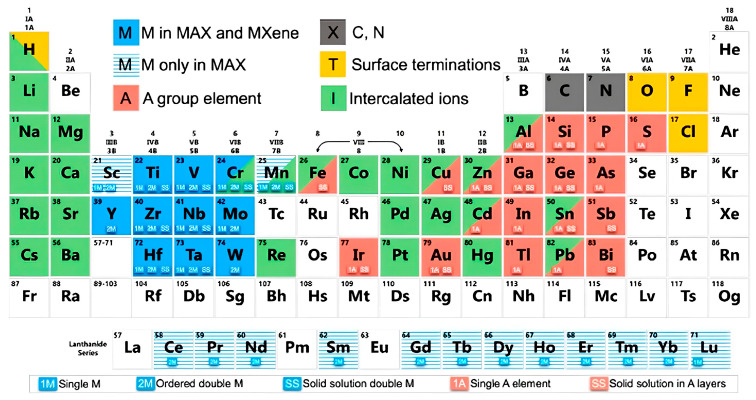
Types of MXene terminations and their advantages. Reproduced from [24], Materials, MDPI.

**Figure 2:**
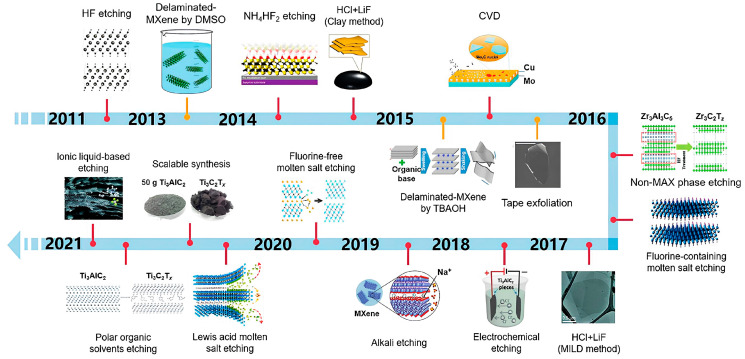
Evolution of MXene synthesis techniques [21].

Synthesis methods for MXenes fall into two primary categories: top-down and bottom-up approaches. Top-down methods typically involve chemical etching techniques, including direct hydrofluoric acid (HF) treatments, Alkali-based reactions, and molten salt approaches. Bottom-up strategies encompass more controlled growth techniques, such as chemical vapor deposition (CVD) and pulsed laser deposition [22]. The choice of etching agent significantly influences the resulting surface chemistry, with HF, HCl, and LiF producing different termination profiles that directly affect the material’s properties. Recent work by Wei et al. [21] has established important correlations between the binding energies of A atoms in MAX phases and their etching behavior, enabling better prediction of MXene formation. Complementary atomic-scale investigations by Ibragimova et al. [23] have systematically characterized surface functionalization in various Ti- and Nb-based carbides and nitrides, though further research is needed to fully understand and control termination effects. Figure 1 below shows elements common to MXene compositions, and Figure 2 shows the evolution of MXene synthesis techniques.

### 2.2. Mechanical, Thermal, and Electrical Properties

Recent investigations into MXenes’ mechanical properties have revealed exceptional characteristics that make them particularly suitable for tribological applications. Comparative studies of Ti_3_C_2_T_x_ and Nb_4_C_3_T_x_ demonstrate their superior mechanical performance relative to other two-dimensional materials, as evidenced by their mechanical strength and Young’s modulus measurements (Table 1). The remarkable mechanical properties of MXenes stem from their unique layered structure, which combines strong in-plane covalent bonds with weak interlayer van der Waals interactions [25]. This structural configuration enables easy interlayer sliding while maintaining structural integrity under applied loads, making them exceptionally resistant to wear in tribological systems. Their high tensile strength ensures durability under stress, while the substantial elastic stiffness provides dimensional stability during mechanical deformation. These properties collectively contribute to MXenes’ outstanding performance as solid lubricants, where they outperform conventional materials like graphene and MoS_2_ in terms of both mechanical resilience and frictional characteristics. Table 1 provides a comparison of mechanical properties of MXene materials with other 2D materials.

The thermal behavior of MXenes is equally remarkable and composition-dependent, with surface terminations playing a critical role in their thermal stability. Fluorine-terminated MXenes exhibit initial decomposition at approximately 550 °C [22], corresponding to the thermal stability limit of fluorine groups. Beyond this threshold, oxygen incorporation occurs at exposed surface sites, followed by progressive oxidation around 1200 °C. Complete structural degradation typically occurs near 1800 °C [22], although these thresholds vary depending on the specific MXene composition and environmental conditions. This thermal stability profile, combined with their excellent thermal conductivity (one study found up to 68.5% improvement by incorporating MXenes) [28], makes MXenes particularly valuable for high-temperature tribological applications where conventional lubricants fail. The materials’ ability to maintain structural integrity across a broad temperature range while facilitating heat dissipation from frictional interfaces significantly enhances their potential for aerospace and advanced manufacturing applications. Furthermore, MXenes exhibit exceptional electrical conductivity (reaching up to 34,500 S/cm for Ti_3_C_2-n_N_X_T_Z_ films) [4], enabling their use in applications requiring simultaneous electrical conduction and lubrication. This unique combination of mechanical robustness, thermal stability, and electrical conductivity positions MXenes as versatile, multifunctional materials capable of addressing complex engineering challenges in extreme environments.

## 3. Tribological Performance of MXenes

### 3.1. MXenes as Solid Lubricants

Perhaps the clearest application for MXenes in Tribology is their use as solid lubricants. Their layered structure, low shear strength, and tunable surface chemistry offer tribological properties that are comparable to or even superior to conventional solid lubricants such as graphite, molybdenum disulfide (MoS_2_), and polytetrafluoroethylene (PTFE) [29]. Recent experimental work has demonstrated the practical benefits of using MXene Ti_3_C_2_T_x_ nanosheets as a solid lubricant in machine elements. When applied as coatings in thrust ball bearings under dry operating conditions, MXene-coated bearings exhibited a reduction in frictional torque by a factor of 3.2 compared to uncoated references. Additionally, these coatings extended service life by approximately 2.1 times and reduced wear rate by up to 2.9 times [29]. These results are on par with established systems such as graphene and transition metal dichalcogenide coatings, demonstrating MXenes’ potential as solid lubricants. In journal bearing applications, MXene coatings also showed improved wear resistance and friction reduction under varying humidity levels. In particular, MoS_2_ demonstrated impressive performance, achieving an 11-fold increase in cycles to failure relative to uncoated references [30]. These results are summarized in Table 2 below.

The lubrication mechanisms of MXenes are attributed to their ability to form low shear tribofilms. Coating thickness and contact pressure are critical factors in solid lubrication, as they influence tribofilm formation. Too thin a coating lacks sufficient material for lubrication, while too thick a coating can hinder effective film formation due to competing exfoliation. Testing showed that higher contact pressures reduced friction, while coating thicknesses below the surface roughness increased it [30]. Environmental factors, particularly humidity, also play a critical role by influencing the intercalation of water molecules and subsequent changes in the formation and durability of the tribofilm [30]. Table 3 shows MXene performance measured experimentally, and Table 4 shows MXene stability as measured computationally.

### 3.2. Friction and Wear Behavior in Various Environments

The tribological performance of MXenes is highly sensitive to the environmental conditions under which they are applied. To account for this, MXene-based materials must be tailored to the specific conditions they will encounter, as their tribological behavior is heavily influenced by factors such as humidity, temperature, vacuum, and submersion in water.

In humid environments, MXenes tend to undergo oxidation, which results in increased friction and wear. Although elevated humidity is known to be detrimental to tribological behavior, no optimal MXene configuration has been conclusively identified to mitigate these effects in such conditions [38]. Similarly, high-temperature environments exacerbate oxidation, thereby intensifying wear and friction. However, the use of EB nanosheet composites has been shown to provide better performance at elevated temperatures by resisting oxidative degradation [39]. Though the risk of oxidation in vacuum environments is greatly reduced, the benefits of MXenes are still dampened compared to ambient conditions. The effectiveness of MXenes in vacuum is notably enhanced when combined with other materials. For instance, MXene/MoS_2_ hybrids have demonstrated improved friction and wear behavior compared to either MXenes or MoS_2_ alone [40]. MXene additives can improve the tribological performance of oil-based lubricants. The addition of small amounts of MXene (less than 0.1 wt.%) has been shown to enhance lubrication properties [41]. In aqueous systems, the behavior of MXenes varies greatly depending on their specific structural and chemical configuration. For example, MXene/graphene oxide (GO) composites have shown promise in maintaining low friction and wear in water-based environments due to synergistic effects between the two materials [42]. Table 5 below summarizes these findings.

This comprehensive analysis reveals MXenes as environmentally adaptive materials whose tribological properties can be precisely tuned through surface engineering and composite design, offering opportunities for optimization across diverse industrial sectors. The development of advanced characterization techniques and computational modeling approaches continues to deepen our understanding of these structure-property relationships, enabling more sophisticated designs for demanding operational environments. Future research directions should focus on expanding the environmental limits of MXene performance while addressing challenges related to long-term stability and large-scale production feasibility. These challenges are further discussed in Section 8 of this review.

### 3.3. Role of Surface Terminations on Tribological Properties

The exceptional tunability of MXenes’ surface chemistry represents a transformative advantage in tribological applications, where specific terminations (e.g., -OH, -O, -F) precisely control interfacial behavior and lubrication mechanisms. These termination groups serve as molecular levers that manipulate adhesion force, shear resistance, and environmental stability. -O terminations have low shear stress, making them well-suited to use in solid lubrication [16]. In contrast, -OH and -F terminations resist sliding more strongly [16]. The inclusion of strong hydrogen bonds leads to -OH having greater resistance to failure, though -O terminations are generally best at lower temperatures and pressures [43]. Advanced termination engineering has enabled the development of hybrid surfaces (like -O/-Cl mixtures) that combine the benefits of multiple functional groups [44]. The manipulation of these surface chemistries allows exceptional customization potential for MXene tribological performance. Beyond influencing friction and wear, surface terminations also have a strong impact on the thermal, electronic, and chemical characteristics of MXenes. For instance, -F terminations have been shown to increase thermal conductivity relative to -O and -OH terminations, making them potentially useful in high-heat environments where thermal dissipation is critical [44]. Although -F groups typically reduce mechanical strength and lubricating performance [16,43], their presence may benefit applications where thermal management outweighs mechanical considerations. Table 6 below compares the performance of these surface terminations.

## 4. Applications of MXenes in Tribology

### 4.1. Lubrication in Micro and Nano-Scale Systems

The emergence of micro and nano-scale electromechanical systems (MEMS/NEMS) has revolutionized modern technology, enabling compact, energy-efficient solutions across diverse sectors, including consumer electronics, medical devices, automotive systems, and aerospace applications [45]. These miniaturized systems, while technologically transformative, face significant tribological challenges due to their silicon-based components’ inherent wear susceptibility and high surface-to-volume ratios that amplify frictional losses. MXenes have emerged as a groundbreaking solution for these challenges, offering exceptional lubrication performance at micro/nano-scales through their unique combination of mechanical robustness [5], ultra-low shear resistance, and tunable surface chemistry. The two-dimensional nature of MXenes allows them to form conformal, atomically thin lubricating films that reduce friction coefficients while maintaining remarkable wear resistance, outperforming conventional solid lubricants like MoS_2_ in MEMS/NEMS applications [46]. Particularly promising are MXene composite systems, where ionic bridging between the materials creates synergistic effects. MXenes provide structural stability and load-bearing capacity that can be combined with existing materials, resulting in composite films with exceptional resistance to wear and mechanical yield [46].

The future development of MXene-based lubrication for micro/nano-systems hinges on optimizing synthesis methods to ensure precise control over flake size, surface termination, and defect density [47], all critical parameters that determine tribological performance at these scales. Advanced deposition techniques like molecular layer assembly and electrophoretic deposition are showing promise for creating uniform MXene coatings on complex microstructures. Furthermore, the integration of computational materials design with experimental validation is accelerating the discovery of optimal MXene compositions and hybrid architectures specifically tailored for MEMS/NEMS applications. As research progresses, MXenes are poised to overcome current limitations in miniaturized system reliability, enabling next-generation devices with longer operational lifetimes and reduced energy losses from friction, while their inherent electrical conductivity opens possibilities for smart tribological systems capable of in situ wear monitoring and self-adjusting lubrication.

### 4.2. Additives for Lubricants and Greases

MXenes have also shown promise as additives for lubricants and greases, offering improvements in tribological performance under extreme operating conditions. The unique two-dimensional structure and surface chemistry of titanium-based Ti_3_C_2_T_x_ MXenes make them particularly effective for enhancing conventional lubricating formulations, bridging performance gaps in high-temperature and high-load applications where standard oil-based greases fail. Recent research by Zhang et al. [48] demonstrated these advantages through comprehensive testing of MXene-enhanced lithium grease (MLG), where Ti_3_C_2_ nanosheets were incorporated at varying concentrations (1–4 wt.%) and subjected to rigorous ball-milling for optimal dispersion. Tribological evaluation using a rotating ball-on-disk configuration revealed remarkable improvements, with optimal formulations achieving a 56.7% reduction in friction coefficient and a 26.6% decrease in wear rate compared to baseline grease, attributable to the formation of durable tribofilms that maintain lubrication integrity. Complementary work by Ma et al. [49] further validated these findings in oil-based systems, developing an HCl-LiF etching protocol to produce Ti_3_C_2_T_x_ additives that demonstrated exceptional wear reduction (92% at 100N load) when dispersed in 5750 lubricating oil. The lubrication mechanism relies on MXenes’ ability to form protective, sliding interlayers between contact surfaces. Table 7 below quantifies these performance enhancements across different lubrication systems.

**Figure 3:**
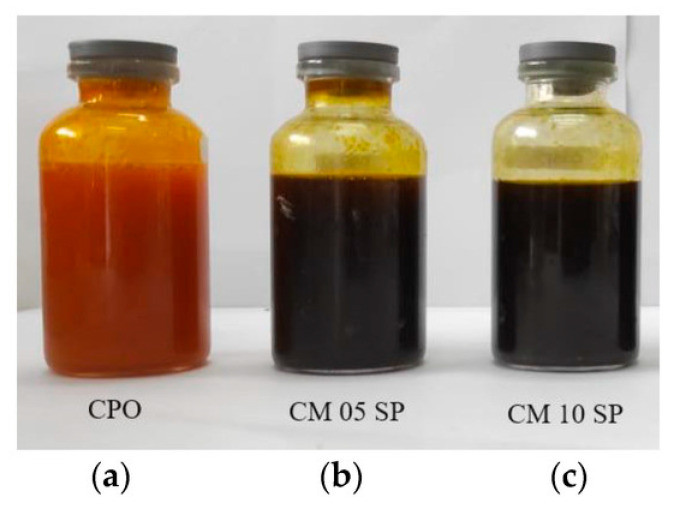
(**a**) Pure crude palm oil (CPO), (**b**) 98% wt. of CPO with 0.5% wt. of CMC and MXene, respectively, and 1% wt. of Span 60 (CM 05 SP), and (**c**) 96% wt. of CPO with 1% wt. of CMC and MXene, respectively, and 2% wt. of Span 60 (CM 10 SP). Reproduced from [51], Lubricants, MDPI.

The industrial adoption of MXene lubricant additives faces challenges, including oxidation stability during long-term storage and the need for optimized dispersion techniques to prevent nanosheet agglomeration. Current research focuses on surface functionalization strategies to enhance MXene compatibility with various base oils and greases, while advanced characterization techniques like in situ TEM tribometry are providing new insights into the real-time formation and behavior of MXene-derived tribofilms. These developments position MXenes as a next-generation additive technology capable of extending equipment service intervals, reducing energy losses from friction, and enabling operation in previously inaccessible temperature-pressure regimes across automotive, aerospace, and industrial machinery applications. Future directions include the development of smart MXene additives capable of pressure-adaptive lubrication and the integration of MXenes with other nano-additives to create synergistic lubrication systems with multifunctional capabilities. Figure 3 shows an MXene composite used in bio-lubrication.

### 4.3. Tribological Coatings and Thin Films

MXene-based coatings represent a significant advancement in surface engineering, offering exceptional tribological performance through innovative composite architectures and material combinations. These two-dimensional materials demonstrate unique capabilities when incorporated into protective films, particularly in systems combining MXenes with polydopamine (PDA) matrices or laser-textured substrates, where they form lubricating tribofilms that maintain integrity under mechanical stress [52]. Research by Chen et al. [52] on Si-MX/PDA-HOAC composite films revealed a breakthrough in coating technology, where the system of MXene nanosheets (providing mechanical reinforcement), PDA (acting as an adhesive binder), and HOAC (forming the lubrication layer) created a synergistic effect that reduced friction coefficients and increased load-bearing capacity relative to each material. Advanced characterization through FESEM, EDS, and XPS analysis identified the formation of FeF_2_-rich tribochemical layers that contribute to the coating’s exceptional wear resistance [52]. Parallel developments in surface texturing engineering, as demonstrated by Zhang et al. [53], show that combining laser surface texturing (LST) with 2D MXene nanocoatings on titanium alloys achieves 70% reduction in friction and a near-elimination of substrate wear through a unique self-replenishing mechanism where frictional forces actively redistribute MXene nanosheets from textured reservoirs to form durable tribofilms. This approach overcomes the traditional limitations of standalone surface treatments by creating a dynamic system that maintains optimal lubrication throughout the component’s service life. Table 8 below compares these advances in MXene coating technologies.

The development of MXene coatings faces several technical challenges. Current research focuses on advanced deposition techniques like molecular layer-by-layer assembly [56] and electrophoretic deposition [57] to achieve uniform, defect-free coatings at industrial scales. Furthermore, the integration of computational materials design with experimental validation is accelerating the discovery of MXene-polymer combinations and surface texturing patterns for specific application requirements [58]. These MXene-based coating technologies are particularly promising for aerospace components, biomedical implants, and precision machinery, where conventional lubrication methods are inadequate. Future directions include the development of stimuli-responsive MXene coatings capable of adapting their tribological properties to changing operating conditions and the creation of multifunctional coatings that combine wear resistance with corrosion protection and thermal management. The continued refinement of these coating technologies promises to enable mechanical systems with extended service lives and reduced energy consumption from frictional losses.

## 5. Expanding the Role of MXene-Based Composites

### 5.1. Polymer-MXene Composites

The rapid evolution of modern technology has driven the demand for advanced materials that combine high electrical conductivity, mechanical robustness, and thermal stability, positioning polymer-MXene composites at the forefront of materials innovation. These composites, formed by integrating two-dimensional MXene nanosheets into polymer matrices, exhibit unparalleled multifunctionality, enabling them for applications such as electromagnetic interference (EMI) shielding, energy storage, and the advent of flexible electronics [59]. A critical technological challenge addressed by these composites is EMI pollution, which disrupts electronic devices and poses health risks. Polymer-MXene composites excel in this domain, leveraging MXenes’ conductivity and layered structure to achieve exceptional shielding effectiveness while minimizing secondary electromagnetic reflections. Recent advancements demonstrate that MXene-composite films can achieve excellent shielding capabilities (EMI SE of ~57 dB) with extremely small film thicknesses (in this case, 9 µm), representing a significant leap over conventional shielding materials [59]. Beyond EMI mitigation, these composites have revolutionized energy storage, particularly in supercapacitors, where polymer-MXene hybrids fabricated via electrolyte-free electrochemical polymerization deliver remarkable performance: 69.5 mF cm^−2^ areal capacitance, 250.1 mWh cm^−3^ energy density, and stability over 10,000 cycles, setting new benchmarks for compact, high-power energy devices [60].

The mechanical and thermal enhancements imparted by MXene polymers further expand their utility. These composite materials can significantly improve mechanical strength, thermal stability, and surface properties [61], enabling their use in extreme conditions. Innovative synthesis techniques, such as in situ polymerization and electrospinning, have been optimized to ensure uniform MXene dispersion, critical for maximizing composite performance [61]. For flexible electronics, stretchable elastomer-MXene nanocomposites have been developed, achieving 49 dB EMI SE at 19.6 vol% MXene loading while maintaining high elasticity and durability, a breakthrough for wearable technology and aerospace applications [62]. Despite these successes, challenges like scalable production, long-term oxidative stability, and cost reduction must be addressed to facilitate widespread industrial adoption. These factors are further discussed in Section 8 of this review. Table 9 summarizes key performance metrics of polymer-MXene composites compared to traditional materials.

Polymer-MXene composites represent a shift in multifunctional material design, offering a unique convergence of electrical, mechanical, and thermal performance that surpasses conventional polymer systems. Their exceptional capabilities in EMI shielding, energy storage, lubrication, and flexible electronics stem from the intrinsic properties of MXenes: high electrical conductivity, tunable surface chemistry, and layered morphology combined with tailored polymer processing strategies. As evidenced by the data in Table 7, these composites not only meet but often exceed the performance metrics of traditional materials across diverse applications. While their potential is undeniable, translating these advances into industrial-scale implementation requires overcoming hurdles related to scalability, long-term stability, and cost-effectiveness. Addressing these challenges will be essential to fully realize the transformative impact of polymer-MXene composites.

### 5.2. Metal Matrix MXene Composites

**Figure 4:**
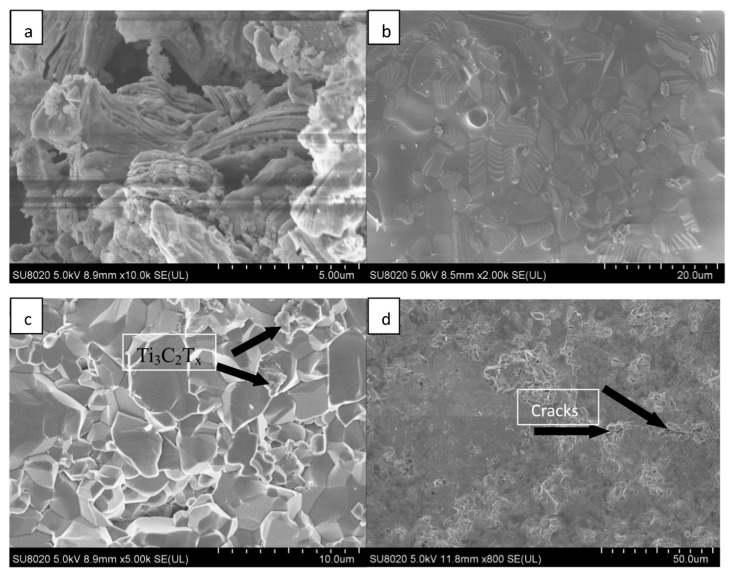
SEM images of Ti_3_C_2_T_x_ (**a**) before and (**b**) after sintering treatment (1400 °C for 90 min); (**c**) cross-sectional surface of sintered Ti_3_C_2_T_x_-Al_2_O_3_ composites; (**d**) the surface of a specimen indented by a load of 5.0 kg. Reproduced with permission from [65].

MXenes have emerged as highly promising reinforcements in metal matrix composites (MMCs) due to their combination of mechanical strength, electrical conductivity, and high-temperature stability [64]. Their integration into metal matrices could yield high-performance structural materials with tunable properties for applications ranging from aerospace to thermal management systems. Unlike conventional reinforcements such as SiC or carbon nanotubes, MXenes offer a unique 2D morphology coupled with high in-plane mechanical strength (up to 386 GPa in the case of Nb_4_C_3_Tx [64]) and excellent surface chemistry for interfacial bonding with metallic matrices. Figure 4 shows SEM images of Metal-MXene composites.

One of the most notable advantages of incorporating MXenes into MMCs is the potential for significant mechanical enhancement at low filler concentrations. For instance, single-flake Ti_3_C_2_Tx MXene has demonstrated up to a 66% improvement in tensile strength in aluminum composites with just 0.2 vol% loading [64]. Such improvements are not limited to aluminum; magnesium-based composites reinforced with size-controlled Ti_3_C_2_Tx MXenes have shown clear trends of increasing compressive yield strength (CYS) with decreasing flake size [66]. Table 10 provides a summary of these improvements.

Another major advantage of MXenes in metal matrices lies in their compatibility with modern processing techniques. Their negative surface charge facilitates solution-based mixing and electrostatic self-assembly [64], techniques which could be vital to the production of MXene composites at scale. This processability allows for homogeneous dispersion in metal powders, critical for uniform property enhancement. Moreover, MXenes maintain structural integrity under moderate sintering conditions. For instance, Ti_3_C_2_Tx in Al matrices remains stable below 923 K, while at higher temperatures it transforms into other composites such as TiC and Al_3_Ti, which may also contribute to mechanical reinforcement [66].

### 5.3. Synergistic Effects with Other 2D Materials

The integration of MXenes with complementary two-dimensional materials has emerged as a strategy to overcome previous limitations while improving multifunctionality in advanced material systems. By combining MXenes with graphene, carbon nanotubes (CNTs), layered double hydroxides (LDHs), and other nanomaterials, researchers have created hybrid architectures that exhibit superior performance across electromagnetic, biomedical, structural, and energy applications [68,69,70]. In electromagnetic wave absorption, carefully engineered heterostructures like Co nanochain-decorated Ti_3_C_2_T_x_ MXenes achieve remarkable shielding effectiveness (−46.48 dB reflection loss at just 1.02 mm thickness), outperforming conventional materials through optimized impedance matching and multiple scattering mechanisms [71]. The biomedical field has particularly benefited from MXene-graphene hybrids, which combine exceptional electrical conductivity (~34,500 S/cm) [4] with antimicrobial efficiency (>99% against pathogens) and photothermal conductivity: properties that proved vital during the COVID-19 pandemic for developing advanced protective equipment and diagnostic platforms [72].

One study from Jin et al. found that structural integrity is also improved with composites. Incorporating a hybrid of MXene and graphene into a PEG polymer matrix led to dramatic improvements in mechanical performance. The resulting composite (MGPP100-3) achieved a 6 times increase in tensile strength, 4 times increase in Young’s modulus, and 9 times increase in toughness compared to pure PEG [73]. These enhancements are attributed to the synergistic reinforcement effect of MXene and graphene, along with strong interfacial bonding facilitated by the polydopamine layer, which ensures efficient stress transfer and structural integrity throughout the composite. Energy storage systems also achieve gains through MXene-LDH heterostructures, where they have been shown to yield supercapacitors with greater structural stability and better long-term cycling ability [74]. Finally, nitrogen-doped MXene hybrids have revolutionized electrocatalysis, achieving hydrogen evolution reaction (HER) performance metrics that rival precious metal catalysts. Table 11 below quantifies these enhancements across various application domains.

## 6. Experimental Techniques in Tribological Studies of MXenes

### 6.1. Measurement of Friction and Wear

**Figure 5:**
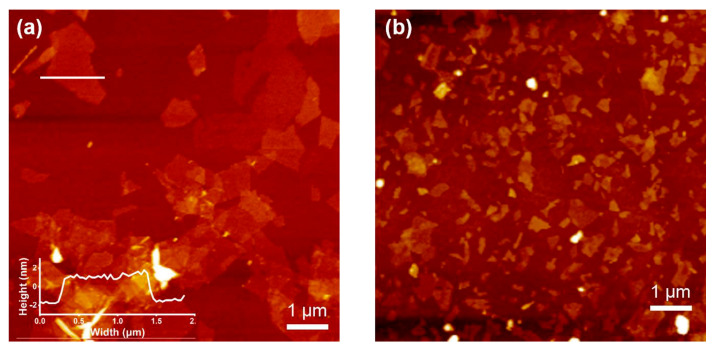
AFM images of Ti_3_C_2_T_x_ MXene: (**a**) Ultrasound 10 min and (**b**) ultrasound 60 min. Reproduced from [78], Polymers, MDPI.

The measurement of friction and wear in MXenes requires careful adaptation of protocols to account for their unique layered and chemically dynamic structure. Unlike most uniform materials, MXenes exhibit complex behavior arising from their 2D flake morphology, surface terminations, and sensitivity to environmental conditions, all of which can strongly influence how they react under mechanical load. Standard tribological testing methods must be adapted to resolve nanoscale effects that dominate their behavior. For example, atomic force microscopy (AFM) and friction force microscopy (FFM) have been used to measure friction at the single-flake level [76,77]. These techniques reveal that friction in MXenes is highly dependent on surface chemistry. Figure 5 below shows AFM images of MXenes.

Thermal annealing, which reduces surface -OH groups, has been shown to decrease friction by over 50% [13]. Furthermore, MXenes’ ability to intercalate ions or small molecules introduces variability in their interlayer spacing, which in turn alters shear response [13]. The structure and chemistry of the flakes evolve over time or under sliding conditions, making real-time measurements critical. Also, MXenes can be susceptible to oxidation [13,17], so measurements must be taken under specific conditions to ensure consistency and repeatability. Tribological testing should occur in dry, humid, and oxygen-free environments to obtain a full range of behaviors. Lastly, to account for MXenes’ tendency to restack or agglomerate, testing systems must ensure homogeneous flake distribution, particularly in lubricated conditions or when used as coatings [13]. Table 12 lists the different experimental techniques for tribological studies and their MXene-specific considerations.

### 6.2. Surface Characterization Techniques

The comprehensive evaluation of MXenes’ tribological performance necessitates advanced surface characterization techniques due to their unique chemical structure. High-resolution scanning electron microscopy (SEM) serves as a cornerstone technique, providing precise visualization of wear, delamination, and debris: critical for assessing failure mechanisms in MXene coatings and composites [79]. Transmission electron microscopy (TEM) extends these observations to the atomic scale, where high-angle annular dark-field (HAADF) imaging and selected-area electron diffraction (SAED) have uncovered stress-induced amorphization at MXene edges and the formation of nanocrystalline TiC domains under severe plastic deformation [80]. These structural transformations, first observed by TEM tribometry, directly correlate with MXenes’ friction-reducing capabilities through the generation of lubricious tribofilms [81].

Chemical characterization techniques play another pivotal role in decoding MXene tribology. In a study by Rodriguez et al., the Ti_2p_ XPS spectra revealed that 62% of the signal arose from titanium carbide, 17% from C-Ti-OH, and 21% from C-Ti-O [77]. These mixed surface terminations significantly influence the frictional properties of the nanosheets by modifying surface energy and potential chemical interactions at the interface. FFM experiments demonstrated that few-layer Ti_3_C_2_T_x_ nanosheets reduced friction by approximately 65% compared to the bare SiO_2_ substrate [77]. Although this performance was somewhat inferior to well-known solid lubricants like graphene and MoS_2_, the reduction was still substantial. The frictional behavior displayed a non-linear load dependence, with a plateau at low loads followed by increased friction at higher loads. Notably, the study proposed that the friction reduction may not solely result from interlayer sliding but could also be attributed to reduced chemical interactions between the FFM tip and the surface, as well as surface roughness effects and intercalated water molecules influencing phonon dissipation mechanisms [77].

In contrast, Chhattal et al. focused on multi-layer V_2_CT_x_ coatings applied to steel substrates and investigated their lubrication performance under prolonged sliding conditions [81]. XPS analysis of the V_2_CT_x_ powder identified multiple oxidation states of vanadium, as well as carbon and oxygen environments corresponding to V-C, C-C, C-C-O, and CO_3_ species. These results confirmed the presence of diverse surface terminations, including -O, -OH, and -F, and indicated slight oxidation during tribological testing. After sliding, additional XPS spectra from the wear tracks and counterbody showed an increase in oxide and amorphous carbon species, suggesting tribo-induced oxidation and degradation of the MXene layers [81].

Together, these studies demonstrate how advanced surface characterization techniques like XPS, AFM, and FFM can reveal the critical roles that surface chemistry, layer structure, and tribochemical transformations play in governing the lubrication performance of MXenes. Table 13 below summarizes key surface characterization techniques for MXenes.

### 6.3. High-Temperature and Harsh Environment Performance

MXenes, particularly Ti_3_C_2_T_x_, have been extensively studied for their tribological performance under varying environmental conditions, including high temperatures and harsh environments. These 2D materials, owing to their unique layered structure, surface chemistry, and mechanical properties, show promising results when incorporated into lubricants as additives, significantly enhancing performance in demanding tribological applications.

MXenes, particularly Ti_3_C_2_T_x_, have demonstrated significant improvements in lubrication performance under elevated temperatures. In several studies, it was observed that adding Ti_3_C_2_T_x_ MXenes to lubricants reduced friction and wear, even under extreme conditions (e.g., temperatures up to 150 °C) [82]. For example, Ti_3_C_2_T_x_ MXenes exhibited a notable reduction in the coefficient of friction (COF) by about 30% when used as an additive in poly-(α)-olefin oils at elevated temperatures [82]. The layered structure of MXenes allows them to form a self-lubricating tribofilm [82], which effectively separates metal surfaces and prevents direct contact, thereby reducing wear. Furthermore, studies have shown that MXenes can improve the oxidation resistance of lubricants [50], making them viable for long-term use under harsh, thermally stressful conditions.

MXenes have also shown exceptional performance under other harsh conditions, such as high humidity or aggressive chemical environments, thanks to their ability to form stable tribofilms. Their surface terminations (e.g., -OH, -O, -F) interact with lubricants and metal surfaces, improving adhesion and wear resistance [83]. In marine environments, for example, MXenes can be incorporated into engine oils, improving the oil’s thermal conductivity and reducing friction and wear significantly [50]. These materials are especially useful in outboard engines, where the lubrication system is often exposed to both high temperatures and corrosive seawater [50].

Additionally, MXenes have shown promise in improving the wear resistance and lubrication of polymer composites used in aerospace and automotive industries. For instance, when integrated into polyimide (PI) composites, MXenes helped to reduce friction and wear, even in extreme conditions like high temperatures [83]. These results indicate that MXenes can provide a dual benefit of reducing friction and enhancing material durability, even when exposed to continuous mechanical stresses and high-temperature environments. Table 14 summarizes these findings.

## 7. Computational Studies and Modeling in MXene Tribology

### 7.1. Density Functional Theory (DFT) Studies on MXene Interactions

Density Functional Theory (DFT) has emerged as an indispensable tool for unraveling the atomic-scale mechanisms governing MXenes’ tribological behavior, providing critical insights that guide material design and optimization. These simulations have revealed that surface terminations (-F, -O, -OH, -Cl) dictate interfacial interactions by modulating adhesion energies and sliding potentials between MXene layers and contacting surfaces. For instance, DFT calculations demonstrate that hydroxyl-terminated MXenes exhibit strong interfacial adhesion (≈1.51 J/m^2^), while uniform terminations have comparatively weaker bonding [16]. The potential energy surfaces (PES) of mixed-termination systems show even more complex behavior, where strategic termination patterning creates energy barriers that facilitate controlled shear while maintaining sufficient adhesion for coating durability [16], a balance crucial for applications like aerospace bearings. Dispersion-corrected DFT methods (e.g., D3-BJ) have been particularly effective in modeling these systems by accurately capturing the interplay between chemical bonding at termination sites and long-range adhesion forces between MXene sheets [84].

Beyond tribology, DFT has illuminated termination-dependent electronic properties across MXene variants. In superconducting Nb_2_C systems, simulations predicted and experiments confirmed that Cl terminations enable superconductivity (Tc ≈ 5.2 K), while their -F terminated counterparts remain unstable [85]. Similarly, for Cr_2_C MXenes, DFT identified Kubas-type hydrogen binding sites with ideal adsorption energies (0.1–0.4 eV/H_2_), enabling reversible 6.4 wt% storage capacity that surpasses DOE targets [86]. These findings underscore DFT’s role as a predictive engineering tool for tailoring MXenes to unique applications.

The predictive power of DFT is further exemplified in studies of MXene heterostructures, where it can provide insight into properties such as the material’s electric field, polarization, and interfacial adhesion [87]. Recent advances combining DFT with machine learning enable high-throughput screening of thousands of termination configurations, accelerating the discovery of MXene formulations with hyper-specific tribological and electronic properties [88]. As computational resources expand, these approaches will play a pivotal role in realizing MXenes’ full potential, from atomically engineered solid lubricants to multifunctional energy materials.

### 7.2. Modeling of Tribological Behavior at Macro and Nano Scales

Understanding and modeling the tribological behavior of MXenes requires a multiscale approach due to their diverse applications ranging from MEMS to bulk tribological coatings. At macro and nano scales, the friction, wear, and lubrication characteristics of MXenes such as Ti_3_C_2_T_x_ are critically influenced by structure, surface terminations, and environmental factors.

At the nanoscale, AFM and lateral force microscopy (LFM) have become essential tools for investigating friction and wear behaviors of 2D materials. Kozak et al. [31] utilized LFM to quantify the friction forces of mono- and double-layer Ti_3_C_2_T_x_ MXenes, demonstrating a coefficient of friction (COF) of 0.087 ± 0.002 for monolayers and 0.082 ± 0.003 for double layers under ambient conditions. These variations are attributed to differences in layer stacking, surface contamination, and the nature of the terminating groups (-O, -OH, -F).

Modeling frictional behavior at this scale requires going beyond classical laws such as Amontons’ law. Kim et al. highlighted the inadequacy of Archard’s wear equation at the nano scale, noting that wear volume does not scale linearly with applied load or inversely with hardness [89]. Instead, wear at the nanoscale is more accurately modeled as an atom-by-atom attrition process governed by thermally activated bond-breaking events. This behavior is well-described by an Arrhenius-type model: $\frac{\partial h}{\partial t}=bf_{a}exp(-\frac{E_{a}}{k_{B}T})$ [89].

Macroscale modeling of MXenes often incorporates traditional tribological laws with modifications to account for their layered structure and compositional tunability. Experimental studies using ball-on-disk tribometers have shown that bulk Ti_3_C_2_ coatings (∼200 nm thick) exhibit significant reductions in both friction and wear rates: up to 4 and 10 times lower, respectively, compared to uncoated substrates [31]. Key to macroscale performance is the ability to tune interlayer interactions through surface termination engineering. Zhang et al. demonstrated that theoretical substitutions of -O with -OH or -OCH_3_ could reduce interlayer COF by up to a factor of 2 (e.g., from 0.24–0.27 to 0.10–0.14) [31,90], suggesting that surface chemistry plays a critical role in large-scale tribological response.

Additionally, tribocorrosion modeling at the macroscale is essential for real-world applications. Hoque et al. [91] emphasized that traditional additive approaches to wear and corrosion are insufficient for MXenes and similar materials. Instead, tribocorrosion must be treated as a synergistic process where electrochemical and mechanical wear interact non-linearly. Mechanistic and synergistic models were proposed to quantify material loss during such coupled degradation. Table 15 below summarizes these methods of characterization.

Current literature suggests that a universal tribological model for MXenes must bridge classical macroscale laws with nanoscale mechanisms like atomic attrition and interfacial adhesion modulation. The complexity of their surface terminations, chemical environments, and morphological variability further requires hybrid modeling frameworks that integrate atomistic simulations, continuum mechanics, and electrochemical effects.

## 8. Challenges and Limitations in MXene Tribology

### 8.1. Stability and Scalability of MXene Materials

Although MXenes were once considered environmentally unstable, recent research shows that this characterization is outdated. Modern synthesis and storage approaches have greatly improved their ambient durability. Studies have demonstrated that Ti_3_C_2_T_x_ MXene thin films can remain chemically and structurally stable in air for more than a decade [17]. This air stability arises because oxidation in dry oxygen is kinetically hindered and thermodynamically unfavorable at room temperature. Fang et al. [92] further revealed that oxidation typically initiates through confined interlayer water rather than direct oxygen attack. When water is trapped between MXene layers, it can hydrolyze and produce reactive oxygen species that oxidize the surface; when this water is removed, Ti_3_C_2_T_x_ remains stable under ambient conditions. Consequently, oxidation becomes significant only at elevated temperatures or under tribological contact, where local heating and moisture accelerate reactions. Similarly, hydrolysis in aqueous dispersions occurs slowly, and high-quality Ti_3_C_2_T_x_ MXenes can remain stable in water for up to ten months [93].

While ambient oxidation is no longer a major limitation, scalability remains a significant barrier to widespread use of MXenes. Conventional hydrofluoric acid (HF) etching introduces safety, environmental, and cost challenges that hinder industrial adoption. Safer alternatives such as LiF/HCl in situ HF etching, molten-salt synthesis, and electrochemical exfoliation have emerged as promising routes, though they still require optimization to ensure consistent termination chemistry, yield, and layer quality [94]. Further improvements in precursor purification, process automation, and green etching strategies will be essential for achieving large-scale, cost-effective, and environmentally responsible MXene production. Table 16 below summarizes these challenges to MXene tribology.

### 8.2. Hydrolysis and Oxidation: Mechanisms of MXene Degradation

MXene degradation arises primarily from two mechanistically distinct processes: hydrolysis and oxidation. These processes differ in their environmental triggers and outcomes.

Hydrolysis occurs in aqueous or humid environments where water molecules intercalate between MXene layers and react with surface titanium to form TiO_2_ while releasing hydrogen gas. This process is thermodynamically favorable in water but can be significantly mitigated in controlled pH or organic solvents. Even so, modern synthesis routes have achieved aqueous stability of Ti_3_C_2_T_x_ dispersions for nearly a year before observable degradation [93].

Oxidation, in contrast, primarily involves the reaction of MXenes with gaseous oxygen; however, Fang et al. demonstrated that this process is not initiated by oxygen itself under ambient conditions but by the presence of confined interlayer water [92]. When water molecules remain trapped between Ti_3_C_2_T_x_ flakes, they can hydrolyze and produce reactive oxygen species that subsequently oxidize the MXene surface, leading to TiO_2_ formation. By removing this confined water, the authors achieved long-term air stability of Ti_3_C_2_T_x_, confirming that oxidation in dry environments is kinetically and thermodynamically hindered. Consequently, direct oxidation in air is largely inactive at room temperature and becomes relevant only under elevated thermal or tribological conditions where local heating or residual moisture facilitates interlayer reactions. This tribo-induced oxidation differs from environmental degradation because it is localized, transient, and often self-limiting once a thin, passivating TiO_2_ tribofilm forms on the contact interface [92].

Understanding these two pathways is essential for designing MXenes suited to specific tribological environments. Air-stable MXene films are appropriate for dry or vacuum systems, whereas water-exposed systems require hydrolysis-resistant terminations or protective coatings. Ongoing work on surface re-termination (for example, -Cl, -S, or -N functionalization) and barrier coatings such as atomic-layer-deposited oxides or ionic-liquid encapsulants shows strong promise for enhancing performance in humid or aqueous conditions [95,97,98].

Therefore, rather than characterizing MXenes as environmentally unstable, the literature now supports a more nuanced view: MXenes are stable under ambient and dry conditions, and degradation is limited to specific hydrolytic or friction-induced scenarios. Future studies should report environmental context explicitly and use in situ characterization to distinguish between these degradation modes.

### 8.3. Challenges in Large-Scale Applications

Scaling up the production of MXenes from laboratory to industrial scale presents unique challenges that slow their commercial implementation. Chief among these is the scalability of current synthesis methods, particularly those involving toxic etching agents like hydrofluoric acid (HF). While HF etching remains the most widely used and efficient technique for producing high-quality MXenes, it carries substantial safety and environmental risks that are exacerbated at industrial volumes [99]. These include the need for cautious handling protocols, specialized waste treatment systems, and protective infrastructure, all of which drive up production costs and limit process scalability [99]. Alternatives such as modified acid etching with LiF and HCl or molten salt methods have been explored, offering improved safety profiles [99]. However, they introduce their own limitations, such as increased process complexity, high energy demands, or lower yields [94], which currently prevents them from replacing HF etching as the go-to method of MXene synthesis.

The selection and quality of precursor materials further complicate large-scale MXene production. The performance and consistency of the final MXene products are highly sensitive to the composition, purity, and availability of the MAX phase precursors used [99]. Impurities or compositional variations in these precursors can lead to inconsistencies in MXene structure and surface chemistry, negatively impacting performance in target applications such as supercapacitors or batteries [99]. As such, large-scale applications of MXenes remain constrained by the need for safe, cost-effective, and reproducible manufacturing methods that also preserve the unique properties that make these materials so promising in the first place. Common MXene production challenges are outlined in Table 17 below.

## 9. Future Directions and Opportunities

### 9.1. Innovations in MXene Functionalization for Improved Tribology

The future of MXenes in tribology largely depends on advanced functionalization strategies that harness unique surface chemistries to create adaptive, high-performance lubrication systems. For instance, recent studies have shown that vacuum annealing can increase electric conductivity by over 10 times [100], and surface chlorination can improve lubrication in high humidities [101]. Integrating MXenes with ionic liquids has also emerged as a promising approach, where tailored cation and anion combinations (e.g., [EMIM], [BMIM]) form nanostructured layers on the MXene’s surface to further increase material strength [102,103]. Hybrid systems combining MXenes with 2D materials like graphene exhibit remarkable synergistic effects. For instance, MXene-graphene composites can reduce friction by ≈37% and, further, reduce wear by 50% [104].

Looking ahead, three transformative opportunities are poised to redefine MXene tribology: (1) The development of stimuli-responsive “smart” MXenes that dynamically adjust surface chemistry in response to temperature, pressure, or electric fields through incorporated shape-memory alloys or piezoelectric materials; (2) Bio-inspired hierarchical architectures mimicking articular cartilage, where MXene flakes are embedded in hydrogel matrices to combine solid lubrication with impact absorption; and (3) AI-driven materials discovery leveraging quantum-accurate machine learning potentials to screen millions of potential functionalization combinations. While challenges persist in scaling these innovations, the relatively recent discovery of MXenes means the field is still rapidly evolving, and continued research is likely to overcome many of the current obstacles. The goal remains the creation of MXene-based tribological systems that self-optimize in real-time, offering ultra-low friction coefficients with virtually indefinite wear life under extreme conditions, a capability that could revolutionize everything from wind turbine bearings to artificial joints.

### 9.2. MXenes for Green and Sustainable Tribological Applications

As concerns grow over the environmental impact of traditional lubricants and tribological materials, MXenes have emerged as a compelling solution for green tribology. In recent years, efforts have increasingly focused not only on the use of MXenes in tribological systems but also on how they are synthesized. Green synthesis of MXenes has become central to their implementation in fields such as environmental remediation, energy storage, and biomedicine, where non-toxic interactions are critical [105].

**Figure 6:**
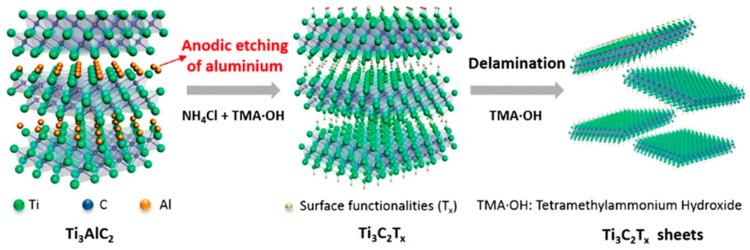
Overview of the HF-free etching process of MXene. Reproduced with permission from [107].

One major pathway to sustainable MXene application involves avoiding hydrofluoric acid (HF), a toxic etchant commonly used in traditional synthesis. HF-based processes pose serious environmental and health risks, such as water pollution [106]. In contrast, green synthesis routes such as electrochemical exfoliation, physical synthesis methods, and HF-free etching offer greener alternatives [106]. Moreover, biologically assisted synthesis (using plant extracts or microbial agents) has gained attention for producing MXenes with enhanced biocompatibility, making them suitable for tribological roles in biomedical implants and environmentally sensitive machinery [106]. Figure 6 details one potential HF-free etching process.

Despite promising developments, scaling green synthesis to industrial levels remains a bottleneck. Challenges such as optimizing reaction parameters, ensuring consistent product quality, and integrating green MXenes into existing lubricant systems require further research and development. Nevertheless, the variety of green synthesis techniques points to a sustainable future for this class of materials. Through ongoing innovations in synthesis and application design, MXenes are poised to support greener engineering systems without compromising technical viability.

Besides their use in oils and other lubricants, MXenes have shown potential as additives for water-based lubricants, which are gaining attention because they are inexpensive, cool surfaces effectively, and are environmentally friendly. The hydrophilic surface groups on MXenes (such as -OH, -O, and -F) allow them to mix and stay well-dispersed in water without needing strong surfactants. This makes them more applicable in aqueous systems than many other 2D materials. When added in small amounts, MXene nanosheets can greatly reduce friction and wear. In one study, Ti_3_C_2_ flakes dispersed in water lowered friction by about 20% and cut wear by 48% compared to pure water [15], while Nb_2_CT_x_ nanosheets reduced friction by roughly 90% and wear by 73% through forming a thin, protective film on the surfaces in contact [108].

However, stability issues must be addressed. MXene nanosheets tend to aggregate or oxidize in water over time, reducing performance. Some studies have observed transformation to TiO_2_ under sliding in humid aqueous environments, which may accelerate wear under certain conditions [42]. To mitigate these effects, strategies such as optimizing flake size, improving oxidation resistance, and using mild stabilizers or surface functionalization are under investigation. Despite these challenges, the existing evidence suggests MXenes are promising eco-friendly nanoadditives for enhancing water-based lubrication. Future research focusing on long-term dispersion stability, oxidation control, and compatibility with water-based formulations will be critical to fully unlock their role in sustainable tribological systems.

### 9.3. Emerging Applications in Space and Extreme Conditions

MXene-based materials are showing strong promise for use in demanding tribological environments such as aerospace and high-temperature systems, due to their combination of structural robustness and excellent electrical and thermal properties. These characteristics make them well-suited for emerging aerospace systems that must endure mechanical wear, extreme temperatures, and electromagnetic interference. MXene films have demonstrated resilience under both temperature extremes, withstanding mechanical stress without significant degradation. Lei et al. developed a flexible MXene/aramid nanofiber composite film that maintained mechanical strengths of 355 MPa at −100 °C and 136 MPa at 300 °C [109]. Not only did this film retain its mechanical integrity, but it also provided exceptional electromagnetic interference (EMI) shielding effectiveness (>99% reduction) under these harsh thermal conditions. This dual performance (in mechanical endurance and EMI shielding) underlines MXenes’ suitability for aerospace applications, where mechanical strength, thermal resilience, and EMI shielding are all essential.

Recent research has shown that MXene-coated fabrics can make composite materials smarter and more useful for demanding environments like space. In one study, an MXene was applied to glass fiber-epoxy fabrics. These coated fabrics worked as built-in sensors, allowing the composite to monitor changes during mechanical strength tests. For example, during the vacuum compaction and resin infusion process, the MXene-coated fabrics detected changes in pressure and resin flow by tracking changes in electrical resistance [110]. This kind of feedback helps manufacturers catch problems early and ensure consistent quality in large or complex aerospace parts. After the composites were made, they were tested under different conditions to see how well the MXene sensors held up. The study found that the sensors could detect bending and strain in the material during mechanical testing [110]. Even after repeated bending, the MXene-coated fabrics continued to send reliable signals. This suggests they could be useful for long-term structural health monitoring in aerospace parts that experience stress over time. Another key finding was in the ability to block electromagnetic interference (EMI), which is important for protecting sensitive electronics in aircraft and spacecraft. MXene-based composites showed better EMI shielding performance than the rGO versions [110]. Table 18 below summarizes MXene performance in extreme conditions.

## 10. Conclusions

MXenes represent a paradigm shift in tribological materials, offering unprecedented versatility through their unique combination of mechanical robustness, thermal stability, and tunable surface chemistry. As demonstrated throughout this review, these two-dimensional composites exhibit exceptional friction-reducing capabilities through their weakly bonded, easily shearable layered structure. Their performance spans diverse environments, from cryogenic space applications to high-temperature industrial systems, where they form adaptive tribofilms that maintain lubrication integrity under extreme contact pressures and thermal cycling. The ability to precisely engineer surface terminations (-F, -O, -OH) via advanced synthesis methods enables targeted optimization of interfacial properties, allowing MXenes to outperform conventional solid lubricants like graphite and MoS_2_ while simultaneously providing multifunctional benefits such as EMI shielding and corrosion protection.

However, the path to commercialization faces significant hurdles that require concerted research efforts. The current reliance on hazardous etchants like HF poses environmental and economic challenges, which currently restrict MXenes to niche applications and industries. Stability concerns, particularly oxidation in air and humidity-induced degradation, demand innovative solutions such as atomic-layer-deposited nanoscale barriers or bio-inspired composite architectures. Perhaps most critically, the transition from laboratory-scale breakthroughs to industrial adoption necessitates standardized protocols for quality control, given that batch-to-batch variations in termination ratios can cause unacceptable fluctuations in tribological performance.

Looking ahead, three transformative opportunities stand out: (1) AI-driven models designed to accelerate the discovery of termination-stabilized MXene formulations, (2) scalable manufacturing techniques like plasma-enhanced dry etching that eliminate liquid waste, and (3) hybrid systems combining MXenes with self-healing polymers or 2D material heterostructures for applications requiring long-term durability. As these innovations mature, MXenes are poised to redefine sustainability in tribology, reducing global energy losses from friction while minimizing environmental impact through longer-lasting components and green lubrication alternatives. With continued interdisciplinary collaboration between computational scientists, chemists, and engineers, the coming decade may witness MXenes transitioning from a scientific curiosity to a cornerstone technology in aerospace, transportation, and renewable energy systems, ultimately fulfilling their potential as the next generation of intelligent, adaptive tribological materials. Future studies should prioritize industry–academia partnerships to bridge the gap between fundamental research and commercial implementation, establishing standardized testing protocols and cost-effective pathways to full-scale industry application.

## Figures and Tables

**Table 1:** Comparative mechanical properties of MXenes and other 2D materials.

Material	Mechanical Strength (GPa)	Young’s Modulus (GPa)	Reference
Ti_3_C_2_T_x_	15.4 (tensile)	484 ± 13	[5]
MoS_2_	23	270 ± 100	[26]
WS_2_	47.0 ± 8.6	302.4 ± 24.1	[27]
WTe_2_	6.4 ± 3.3	149.1 ± 9.4	[27]
Graphene	130	1000	[26]

**Table 2:** MXene performance under thrust ball and journal bearing pressure tests.

MXene Coating	Application	Service Life Improvement Factor	Wear Reduction Factor	Reference
None (Reference)	Thrust Ball Bearing/Journal Bearing	1.0 (Baseline)	1.0 (Baseline)	
Ti_3_C_2_T_x_	Thrust Ball Bearing	2.1	2.9	[29]
Ti_3_C_2_T_x_	Journal Bearing	4	68%	[30]
MoS_2_	Journal Bearing	11	82%	[30]
Ti_3_C_2_T_x_/MoS_2_ (Hybrid)	Journal Bearing	6	68%	[30]

**Table 3:** Experimental MXene performance as a solid lubricant.

Material	COF or Other Metric	Reference
MXenes		
Ti_3_C_2_T_x_ monolayer (on SiO_2_)	0.087 ± 0.002	[31]
Ti_3_C_2_T_x_ double-layer (on SiO_2_)	0.082 ± 0.003	[31]
Ti_3_C_2_T_x_ aqueous dispersion with SDS stabilizer	~213 days of minimal oxidation	[32]
Ti_3_C_2_T_x_ aqueous dispersion buffered with Tris-HCl	Maintained morphology, structure, and good dispersion for ~150 days	[33]
Non-MXene Layered Solids		
Graphite layers sliding in Ultrahigh vacuum	~0.0004	[34]
MoS_2_ nanotube	0.023 ± 0.005	[35]
NiCr/hBN Self-Lubricating Composite	0.28–0.63 depending on temperature	[36]

**Table 4:** Computational MXene stability.

MXene	Exfoliation Energy (via DFT)	Reference
Ti_2_AlC	2.76	[37]
Ti_3_AlC_2_	2.63	[37]
V_2_AlC	3.28	[37]
Nb_2_AlC	2.87	[37]

**Table 5:** Environmental influence on MXene tribological performance and optimization strategies.

Environmental Factor	Tribological Effect	Optimal MXene Configuration	Reference
Elevated Humidity	Causes oxidation, leading to increased friction and wear		[38]
High Temperature	Causes oxidation, leading to increased friction and wear	EB Nanosheet composites	[39]
Vacuum Conditions	Improve performance, but the effect is decreased	MXene/MoS_2_ hybrids	[40]
Oil Lubrication	Enhances tribological properties	Addition of <0.1 wt.% MXene as an oil additive	[41]
Aqueous Systems	Highly dependent on MXene configuration	MXene/GO composite	[42]

**Table 6:** Surface termination effects on MXene performance.

Termination Type	Mechanical Strength	Friction	Key Characteristics	References
-OH dominant	Weaker than -O at low pressure/temperature, but stronger under extreme conditions	Moderate	Strong hydrogen bonds resist sliding	[16,43]
-O dominant	Superior to -OH at low pressure/temperature, but weaker under extreme conditions	Low	Excellent tribofilm potential	[16,43]
-F dominant	Weaker than -O and -OH	High	Superior thermal conductivity	[16,43,44]
Hybrid	Tunable	Tunable	Often arise from standard etching methods	[44]

**Table 7:** Performance metrics of MXene additives in lubricant formulations.

Formulation	Base Medium	Friction Reduction	Wear Improvement	Reference
Conventional	Lithium Grease	Baseline	Baseline	[48]
Ti_3_C_2_T_x_-MLG	Lithium Grease	56.7%	26.6%	[48]
Ti_3_C_2_T_x_-5750	Synthetic Oil	11.2%	92.0%	[49]
Ti_3_C_2_T_x_-ODPA	Supramolecular Gel	46.32%	81.18%	[19]
Ti_3_C_2_T_x_	Outboard Engine Oil	14.5%	6.3%	[50]

**Table 8:** Performance metrics of MXene-based tribological coatings.

Coating System	Substrate	Key Innovation	Friction Reduction	Reference
Si-MX/PDA-HOAC Composite	Silicon	Ternary nanocomposite design	Reduced friction and increased load-bearing capacity	[52]
LST + MXene Nanocoating	Titanium Alloy	Laser-textured reservoirs	Reduced friction by 70% and resulted in minimal substrate wear	[53]
Ti_3_C_2_T_x_	Stainless-steel	Comparison of MXenes to MAX phases	MXene coating surpassed MAX coating in friction reduction by 81.82%	[54]
MXene-Graphene Oxide Composite	Bearing steel	MXene-GO composites	Reduced friction coefficients by significantly more than MXene or GO alone	[55]

**Table 9:** Performance comparison of polymer-MXene composites in advanced applications.

Application	Material System	Key Performance Metrics	Advantages over Conventional Materials	Reference
EMI Shielding	MXene/polymer film	EMI SE 57 dB Film thickness 9 µm	Lower reflection, higher absorption	[59]
Energy Storage	Polymer-MXene composite	69.5 mF cm^−2^; 250.1 mWh cm^−3^; 10,000-cycle stability	Higher energy density, longer lifespan	[60]
Lubrication	Polyimide/V2CTx MXene composite	Wear rate reduced 43.2% at 0.8 wt.% Wear rate reduced 71.9% at 1.2 wt.%	Enhanced durability	[63]
Flexible Electronics	Elastomer-MXene film	EMI SE 49 dB Film thickness 1 mm, High elasticity	Combines flexibility with high shielding	[62]

**Table 10:** Effects of MXene reinforcement on mechanical strength.

Metal Matrix	MXene	Processing Method	Strength Enhancement	Reference
Al	Ti_3_C_2_T_x_	SPS + extrusion	66% (tensile)	[64]
Mg	Ti_3_C_2_T_x_	SPS	17.6% (CYS)	[66]
Cu	Ti_3_C_2_T_x_	Hot pressing	50%	[64]
Mg-Li Alloy	Ti_3_C_2_T_x_	Molten Sonication	128% (tensile YS)	[64]
Al	Ti_3_C_2_T_x_	SPS + extrusion	UTS increased to ~217.9 MPa	[67]

**Table 11:** Performance metrics of MXene hybrid materials versus individual components.

Application	Hybrid System	Key Performance Metric	Improvement over MXene Alone	Reference
EMI Shielding	MXene/Co nanochains	−46.48 dB RL at 1.02 mm, 16.75 GHz	Improved wave absorption	[71]
Biomedical	MXene-Graphene	>99% antimicrobial efficiency	Improved conductivity	[72]
Mechanical Strength	MXene-Graphene	Young’s modulus 5.76 GPa	Improved mechanical strength	[73]
Energy Storage	MXene-NiCo-LDH	High electrical conductivity	Prevented Oxidation	[74]
ORR and HER	MXene and N-doped graphene	Overpotential of 0.36V	Much lower overpotentials	[75]

**Table 12:** Experimental techniques for MXenes from tribological aspects.

Technique	Purpose	MXene-Specific Consideration	Reference
Atomic Force Microscopy (AFM)	Nanoscale friction mapping	Friction responses are sensitive to surface chemistry	[76]
Friction Force Microscope (FFM)	Account for molecular forces during AFM	Friction responses are sensitive to surface chemistry	[77]
Environmental Control	Test under various humidity and temperature conditions	Accounts for thermal and oxidation effects	[13]
Layer-by-layer Coating Analysis	Ensure the durability and consistency of the material	Slight inconsistencies can significantly affect performance	[13]

**Table 13:** Surface characterization techniques for MXene tribology.

Technique	Key Parameters	MXene-Specific Insights	Limitations	References
SEM	Morphology and surface topology	Observe flake structure and film thickness	No chemical composition insight	[81]
TEM	Internal structure, interlayer spacing	Chemical composition	Complex process, limited to small analysis areas	[77,81]
XPS	Surface chemical composition	Identify surface terminations, oxidation states, and surface groups	Surface sensitivity (limited depth profiling), ultra-high vacuum required	[77,81]
AFM	Surface topography, thickness mapping	Measure nanosheet thickness, visualize flake shapes	Limited to small scan areas, tip convolution	[77]
FFM	Nanoscale friction measurement	Nanoscale friction measurement	Influenced by humidity, tip convolution	[77]

**Table 14:** Summary of MXene additives in harsh environments.

MXene Type	Lubricant Base	Key Findings	Reference
Ti_3_C_2_T_x_	Poly-(α)-olefin	Significant friction and wear reduction under high temperatures	[82]
Ti_3_C_2_T_x_	Engine Oil	Improved oxidation and thermal conductivity	[50]
Ti_3_C_2_T_x_	Polyimide Composites	Reduced friction and enhanced wear resistance under high temperatures	[83]

**Table 15:** Methods of MXene tribological characterization.

Scale	Technique/Model	Key Parameters	Findings/Performance Metrics	Reference
Nano	AFM/LFM + Arrhenius model	Surface chemistry	0.082–0.087 COF	[31]
Nano	Archard’s Law	Load, hardness	Does not predict nano wear accurately	[89]
Macro	Ball-on-disk tribometry	Layer thickness, surface terminations	4× reduction in friction, 10× reduction in wear	[31]
Macro	Tribocorrosion modeling	Electrochemical + mechanical wear	Nonlinear wear-corrosion relationship	[91]

**Table 16:** Critical challenges in MXene tribological applications.

Challenge	Technical Impact	Current Mitigation Strategies	Remaining Gaps	References
Surface Degradation	Termination loss due to repeated stress	Molten salt etching	Limited research on differing types of MXenes	[44,95]
Batch Inconsistency	Inconsistency in tribological performance	Advanced process monitoring	Lacking industry standards for QA	[96]
Defect Propagation	Higher wear rates in defective regions	Post-synthesis sorting techniques	Increased production cost	[96]
Scalability Limits	Limited applicability and higher costs	Advanced synthesis approaches	Mitigation strategies still in development	[94]

**Table 17:** Common challenges in large-scale MXene production.

Challenge	Description	References
Toxic Etchants	HF poses severe health risks associated with its handling and disposal. The required precautions increase the cost of manufacturing, and safer alternatives are less efficient.	[94,99]
MAX Precursor Variability	Inconsistent purity reduces MXene quality and performance.	[99]
Oxidation Degradation	Degradation under certain conditions limits shelf and service life.	[94]

**Table 18:** Summary of MXene performance in extreme conditions.

Application	MXene Configuration	Key Functionality	Performance Highlights	Reference
High-temperature environments	MXene/aramid nanofiber film	Thermal stability, EMI shielding, and mechanical strength	Strength 355 MPa @ −100 °C, 136 MPa @ 300 °C, >99% EMI shielding	[109]
Smart sensing in composite manufacturing	MXene-coated glass fiber-epoxy fabrics	In situ process monitoring	Resistance change tracked pressure and resin flow changes	[110]
Structural health monitoring	MXene-coated glass fiber-epoxy fabrics	Strain and bending tracking under mechanical loads	Reliable sensing after 100 flexural cycles	[110]
EMI shielding	MXene-coated glass fiber-epoxy fabrics	Protection of sensitive electronics	Higher EMI shielding than rGO-based composites	[110]

## Data Availability

No new data were created or analyzed in this study. Data sharing is not applicable to this article.
